# ESM-BBB-Pred: a fine-tuned ESM 2.0 and deep neural networks for the identification of blood–brain barrier peptides

**DOI:** 10.1093/bib/bbaf066

**Published:** 2025-02-23

**Authors:** Ansar Naseem, Fahad Alturise, Tamim Alkhalifah, Yaser Daanial Khan

**Affiliations:** Department of Software Engineering, Superior University, 17 KM Raiwind Road Lahore, Punjab 55150, Pakistan; Department of Cybersecurity, College of Computer, Qassim University, Buraydah, Saudi Arabia; Department of Computer Engineering, College of Computer, Qassim University, Buraydah, Saudi Arabia; Department of Computer Science, School of Systems and Technology, University of Management and Technology, Lahore, Pakistan

**Keywords:** artificial intelligence 1, machine learning 2, computational biology 3, Proteomics 4, Computational Biology 5, Bioinformatics 6, Deep Learning 7, blood-brain barrier 8, peptides 9

## Abstract

Blood–brain barrier peptides (BBBP) could significantly improve the delivery of drugs to the brain, paving the way for new treatments for central nervous system (CNS) disorders. The primary challenge in treating CNS disorders lies in the difficulty pharmaceutical agent’s face in crossing the BBB. Almost 98% of small molecule drugs and nearly all large molecule drugs fail to penetrate the BBB effectively. Thus, identifying these peptides is vital for advancements in healthcare. This study introduces an enhanced intelligent computational model called BBB-PEP- Evolutionary Scale Modeling (ESM), designed to identify BBBP. The relative positions, reverse position and statistical moment-based features have been utilized on the existing benchmark dataset. For classification purpose, six deep classifiers such as fully connected networks, convolutional neural network, simple recurrent neural networks, long short-term memory (LSTM), bidirectional LSTM, and gated recurrent unit have been utilized. In addition to harnessing the effectiveness of the pre-trained model, a protein language model ESM 2.0 has been fine-tuned on a benchmark dataset for BBBP classification. Three tests such as self-consistency, independent set testing, and five-fold cross-validation have been utilized for evaluation purposes with evaluation metrics includes accuracy, specificity, sensitivity, and Matthews correlation coefficient. The fine-tuned model ESM 2.0 has shown superior results as compared to employed classifiers and surpasses the existing benchmark studies. This system will support future research and the scientific community in the computational identification of BBBP.

## Graphical Abstract

**Graphical Abstract:**
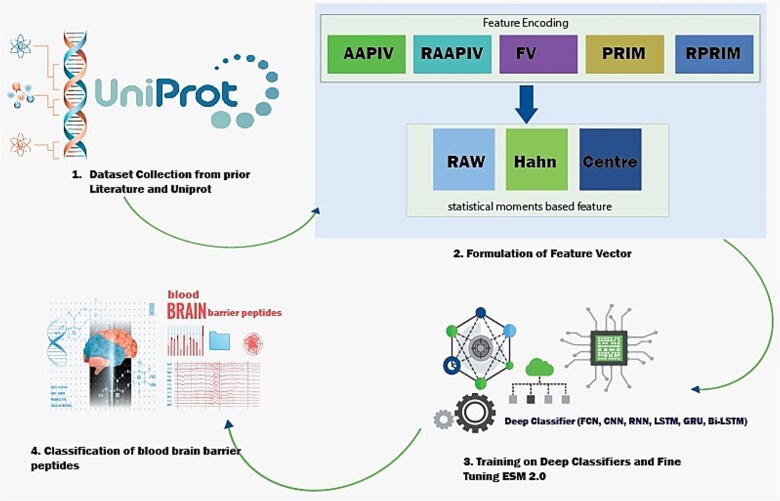

## Introduction

All of the body’s tissues and organs depend on blood arteries for the transportation of oxygen and nutrients [1]. The blood arteries that vascularized the central nervous system (CNS) have specific properties identified as the blood–brain barrier [2]. This barrier controls ion, chemical, and cell flow between the circulation and the brain. By carefully regulating CNS homeostasis, the blood–brain barrier protects neural tissue from damaging poisons and viruses and promotes normal neuronal function. Changes in this barrier’s integrity perform a major function in the development and course of a number of neurological illnesses [3]. Controlling these processes depends on the existence of barrier layers at crucial points where the blood and brain tissue converge [1].

Blood–brain barrier penetrating peptides (BBBPs) can cross the blood–brain barrier via a variety of methods without damaging the structure’s integrity [4]. Certain BBBPs have been shown in studies to promote medication transport into the brain, presenting new possibilities for the creation of treatments for diseases of the CNS [5]. The stagnation in treating CNS disorders arises from the significant challenge of pharmacological drugs crossing the BBB. Almost 100% of large molecule-based medicines and 98% of small molecule-based drugs are unable to effectively penetrate the BBB [6].

The proposed study has made following contributions

1. PRIM, RPRIM, AAPIV, RAAPIV, and FV were computed to construct a feature vector on a collected benchmark dataset.
2. For dimensionality reduction, Hahn, Raw, and central based Statistical moments have been utilized
3. Deep architectures such as fully connected networks (FCN), convolutional neural networks (CNN), recurrent neural networks (RNN), long short-term memory networks (LSTM), bidirectional LSTM (Bi-LSTM), and gated recurrent unit (GRU) have been employed for prediction of BBB peptides.
4. A pre-trained model Evolutionary Scale Modeling (ESM) 2.0 has been fine-tuned on a benchmark dataset.
5. Three tests such as self-consistency, independent set, and five-fold cross-validation (CV) have been utilized for prediction of BBB peptides.
6. Accuracy, specificity, recall, MCC and AUC score have been employed as evaluation metrics for BBB peptides prediction.

There has only been a few research on the computational discovery of blood–brain barrier peptides. A study by Dai *et al.*[7] accomplished a study for identification of BBB peptides that utilizes feature selection approach to eliminate repetitive and irrelevant variables. Finally, Logistic Regression (LR) was used to predict BBB peptides [7]. The next work has extended the dataset and utilized several feature descriptors for feature vector computation [8]. For classification purposes, this study has employed classifiers such as Decision Tree (DT), Random Forest (RF), LR, Support Vector Classifier (SVC), Gaussian Naive Bayes, XGBoost (XGB) and K-nearest neighbor (KNN) [8]. Another study by Chen *et al.* [9] has extended the benchmark dataset and utilizes the feature descriptors such as CKSAAP and PAAC. The classifiers such as DT, KNN, RF, AdaBoost, LogisBoost, GentleBoost, rbfSVM, and linearSVM have been employed for BBB peptides identification [9]. We have contributed to the latest work on BBB peptides prediction by utilizing the ensemble approaches such as bagging classifiers consisting of RF and Extra Trees (ET), boosting consists of LGBM and XGB. Finally, stacking employed XGB and ET utilizes and blending ensemble employed utilizes the LGBM and RF as base learners and LR as Meta learner [10].

## Materials and methods

This section states about the methodology which comprises dataset description, feature vector approaches and classification approaches. The classifier section includes six Deep architectures: FCN, CNN, RNN, Bi-LSTM, LSTM and GRU. Finally, a pre-trained protein language model ESM2.0 has been employed.

**Figure 1:**
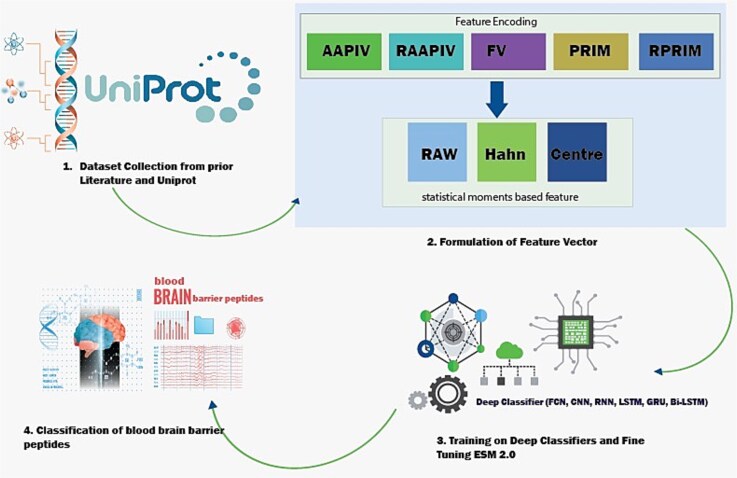
Architecture for BBBPs identification.

Figure 1 depicts the architecture used to identify BBBPs. The relative positioning and statistical moments-based features have been employed. Six deep learning approaches have been used.

### Dataset description

The benchmark dataset was compiled by Chen *et al.* [9]. Several research works, including B3Pdb Kumar *et al.* [11], Dorpe *et al.* [12], and publicly available datasets from BBPpred Dai *et al.* [7] and B3Pred Kumar *et al.* [8], were the data sources of the BBBPs that underwent experimental validation. The peptides related to the blood–brain barrier, brain, Brainpeps, B3Pdb, venom, toxins, trans membrane transport, transfer, membranes, neuro, and hemolysis, were removed from the sequences gathered from UniProt using exact query parameters for the collection of non-BBBPs. Dai *et al.* [7] then used CD-HIT to eliminate redundant sequences with a 10% sequence identity cut-off. Peptide sequences containing ambiguous residues were eliminated. This procedure results in generating 425 non-BBBPs. There are 425 positive and 425 negative samples in the entire dataset. The feature vectors were created from a pooled dataset of positive and negative sequences. To achieve better results, three classifiers’ hyper parameters were tuned over the full dataset. After determining the best hyper parameters, the dataset was split into 77% for training and 23% for testing.

### Feature formulation

Peptides sequences have processed using a feature extraction approach which utilizes composition-specific and position specific features. These popular methods consist of the following components.

### Position relative incidence matrix

The intrinsic properties of proteins are largely dependent on where amino acid residues are positioned throughout the polypeptide chain. A matrix is assembled to capture positional correlations through all residues to highlight intricate patterns established by the arrangement of residues [13]. In order to evaluate the position-specific information of a protein, the 20 unique amino acid residues that are found throughout every polypeptide chain are taken into consideration while creating the PRIM matrix [14].


(1) \begin{equation*} {R}_{PRIM}=\left[\begin{array}{@{}cccccc@{}}{R}_{1\to 1}& {R}_{1\to 2}& \cdots & {R}_{1\to y}& \cdots & {R}_{1\to 20}\\{}{R}_{2\to 1}& {R}_{2\to 2}& \cdots & {R}_{2\to y}& \cdots & {R}_{2\to 20}\\{}\vdots & \vdots & & \vdots & & \vdots \\{}{R}_{x\to 1}& {R}_{x\to 2}& \cdots & {R}_{x\to y}& \cdots & {R}_{i\to 20}\\{}\vdots & \vdots & & \vdots & & \vdots \\{}{R}_{A\to 1}& {R}_{A\to 2}& \cdots & {R}_{A\to y}& \cdots & {R}_{A\to 20}\end{array}\right] \end{equation*}

Every element of the PRIM matrix (Rij) represents the sum which is derived from the ith residue’s relative placement in relation to the jth residue, showing that the ith residue is present at that place. The resultant matrix has 400 coefficients as a result. Statistical moments are computed in order to reduce the dimension complexity; this generates a list of thirty features that are taken out of the 400-coefficient matrix that was initially created.

### Reverse position relative incidence matrix

Similar to the previous enumeration process, the RPRIM method investigates to uncovering hidden characteristics of homologous peptide sequences. RPRIM is calculated using the original sequence’s reverse sequence [15]. The RPRIM matrix that was produced by this process is displayed below.


(2) \begin{equation*} {R}_{RPRIM}=\left[\begin{array}{cccccc}{Q}_{1\to 1}& {Q}_{1\to 2}& \cdots & {Q}_{1\to y}& \cdots & {Q}_{1\to 20}\\{}{Q}_{2\to 1}& {Q}_{2\to 2}& \cdots & {Q}_{2\to y}& \cdots & {Q}_{2\to 20}\\{}\vdots & \vdots & & \vdots & & \vdots \\{}{Q}_{x\to 1}& {Q}_{x\to 2}& \cdots & {Q}_{x\to y}& \cdots & {Q}_{i\to 20}\\{}\vdots & \vdots & & \vdots & & \vdots \\{}{Q}_{A\to 1}& {Q}_{A\to 2}& \cdots & {Q}_{A\to y}& \cdots & {Q}_{A\to 20}\end{array}\right] \end{equation*}

The RPRIM matrix possesses the same dimensionality as the PRIM matrix, with 400 coefficients. Nevertheless, as with PRIM, the feature dimension of RPRIM is decreased to 30 coefficients by using statistical moments.

### Frequency vector

For the purpose of figuring out residue distribution across a polypeptide chain in a specific sequence, the frequency vector has been used as a helpful tool [16]. It calculates the frequency of a given protein’s residue. Protein sequence composition and distribution data are assured due to the FV feature. The FV is depicted in the following.


(3) \begin{equation*} FV = [{f}_1,{f}_2,{f}_3,\dots, {f}_{20}] \end{equation*}

Based on the alphabetic ordinal value of each residue in the peptide sequence, the FV, a 20-dimensional vector, calculates its frequency.

### Accumulative absolute position incidence vector

The FV gathers the distribution-specific information of every residue in amino acid and recognizes perplexing features in a protein’s composition. Conversely, the relative positions of the residues are not recognized by the FV. The development of the AAPIV divided relative position-specific information into four quarters in order to address this problem [17]. The presence of the 20 native amino acids, as listed below, is the basis for this data.


(4) \begin{equation*} K = [{\forall}_1,{\forall}_{2,}{\forall_3}_{,},\dots, {\forall}_n] \end{equation*}

Where the AAPIV’s ${i}_{th}$ segment is determined as


(5) \begin{equation*} {\forall}_i={\varSigma^n}_{k=1}\ {\beta}_k \end{equation*}

Regarding a particular residue, k indicates a random place. The AAPIV’s specified part, represented as I, aggregates all the locations where the ${i}_{th}$nucleotide is present.

### Reverse accumulative absolute position incidence vector

The primary difference between RAAPIV and AAPIV is that it constructs the output vector by utilizing the reverse sequence of the original sample. The reverse facilitates the retrieval of supplementary information on position-specific information, hence allowing the discovery of profound and concealed characteristics within the sequences [18]. The representation of the vector is as follows.


(6) \begin{equation*} RAAPIV = [{n}_1,{n}_{2,}{n_3}_{,},\dots, {n}_m] \end{equation*}

### Statistical moments

The raw, Hahn, and central moments of the genomic data are used to populate the feature set, which contribute key features to the model’s input vector. Researchers have demonstrated that the relative positions and contents of bases in proteomic and genomic sequences influence their characteristics. In order to improve the feature vector, computational and mathematical techniques have been established to capture the correlated arrangement of nucleotide bases in genomic sequences [19]. Having a strong emphasis on associated placement is essential to creating a robust and comprehensive feature set [20].

As Hahn moments necessitate two-dimensional data, genomic sequences are converted into a two-dimensional matrix S′ with dimensions k*k. While this matrix S′ retains the same information as the original matrix S′, consequently.


(7) \begin{equation*} \mathrm{k}=\surd \mathrm{n} \end{equation*}

(8) \begin{equation*} {S}^{{\prime}}=\left|{S}_{11}\ {S}_{12}\dots{S}_{1n}\ {S}_{21\dots }\ {S}_{22\dots}\dots{S}_{2n\dots }\ {S}_{n1}\ {S}_{n2}\dots{S}_{nn}\ \right| \end{equation*}

The generated square matrix is used to compute statistical moments, which produce a fixed-size feature vector and reduce dimensionality.

The raw moments of order a + b can be calculated using the equation below.


(9) \begin{equation*} {U}_{ab}={\varSigma^n}_{e=1}{\varSigma^n}_{f=1}\ {e}^a{f}^b\delta ef \end{equation*}

Important information is embedded in the sequences’ moments, notably up to the third order are${U}_{00},{U}_{10}$*,*${U}_{11}$*,*${U}_{20}$*,*  ${U}_{02}$*,*  ${U}_{21}$*,*  ${U}_{12,}{U}_{03}$ and ${U}_{30}$. To calculate the central moments ($\underset{\_}{x}\underset{\_}{,y}$), to find the central point of the data, first compute the centroid. After that, the central moments are calculated using this centroid and the steps listed below:


(10) \begin{equation*} {v}_{ab}={\varSigma^n}_{e=1}{\varSigma^n}_{f=1}{\left(e-\underset{\_}{x}\right)}^a{\left(f-\underset{\_}{y}\right)}^b\delta ef \end{equation*}

A square grid is the discrete input used to calculate Hahn moments. This method makes it possible to rebuild the original data using inverse Hahn moments, which highlights the regularity and reversibility of the data. The reversibility characteristic of Hahn moments ensures that the information from the original sequences is preserved and incorporated into the model through the feature vector. The following formula illustrates how Hahn moments are calculated.


(11) \begin{align*} {h}_n^{x,y}\left(p,Q\right)= & {\left(Q+V-1\right)}_n{\left(Q-1\right)}_n\times{\varSigma^n}_{z=0}{\left(-1\right)}^z \nonumber \\ & \times \frac{{\left(-n\right)}_z{\left(-p\right)}_z{\left(2Q+x+y-n-1\right)}_z}{{\left(Q+y-1\right)}_z{\left(Q-1\right)}_z}\frac{1}{z!} \end{align*}

The equation makes use of the Gamma operator and Pochhammer notation, are covered in detail by Akmal *et al.* [21].

The Hahn coefficients derived from the previous equation are usually normalized using the coefficients presented in the subsequent equation.


(12) \begin{align*} {H}_{pq} = & {\varSigma^{G-1}}_{j=0}{\varSigma^{G-1}}_{i=0}\ {\delta}_{pq}{h^{a,b}}_p\left(j,Q\right)\ {h^{a,b}}_q\ \left(i,Q\right),\kern1.5em \nonumber \\ & \qquad \qquad \qquad \qquad m,n=0,1,2,\dots, Q-1 \end{align*}

### Classifiers

In this section deep classifiers such as FCN, RNN, CNN, LSTM, Bi-LSTM, and GRU have been utilized. In addition, a fine-tuned protein language ESM 2.0 model on benchmark dataset has been explained.

### Fully connected network

In a fully connected neural network, there are multiple layers, starting with the input layer where the feature vector (X) is provided. After the input layer, each hidden layer contains neurons that are fully connected to every neuron in the subsequent layer. To make predictions, the input feature vector is passed through the network, and activations are computed for each neuron in the hidden layers. These activations are obtained by applying a weighted sum of the inputs feature values from the previous layer, along with bias terms. The result then goes through an activation function, like sigmoid or ReLU, to introduce non-linearity and enable the network to capture complex relationships between features [22].

This process is repeated for each hidden layer until we reach the final layer. The final layer generates the predictions for the given input, represented as Ŷ (Y-hat). To calculate the output Ŷ, we again perform a weighted sum of the outputs from the last hidden layer, along with a bias term, and then apply the activation function to obtain the predicted output. The final predictions (Ŷ) represent the network’s estimation of the target output for the given input data. The formula for Ŷ can be written as:


(13) \begin{equation*}\widehat{Y} = \sigma \left(W(L)\ast A\left[L-1\right]+b\left[L\right]\right) \end{equation*}

Where:

$\hat{\mathrm{Y}}$ is the predicted output vector. $\mathrm{\sigma} \left(\right)$is the activation function applied elementwise to the vector. $\mathrm{W}\left[\mathrm{L}\right]$represents the weight matrix between the last hidden layer $(\mathrm{L}-1$) and the output layer $\mathrm{L}$. $A[\mathrm{L}-1$] denotes the activation vector from the last hidden layer.$\mathrm{b}\left[\mathrm{L}\right]$ is the bias vector for the output layer.

**Figure 2:**
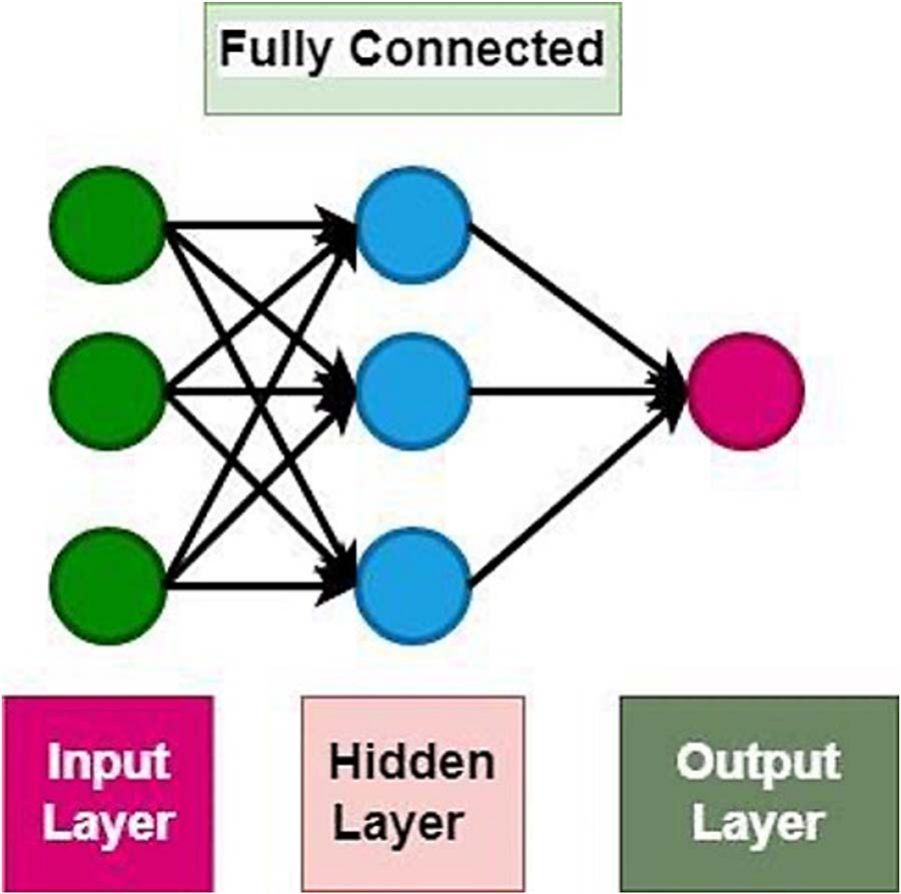
FCN graphical architecture.

Figure 2 depicts the structural framework of the FCN Architecture, which was used to predict BBB peptides.

Table 1 demonstrates the FCN parametric architecture for BBB peptides classification.

**Table 1:** FCN parametric architecture.

Layer	Type	No. of neurons	Total weights
Input	-	N	0
Hidden	Dense	288	N*288 + 288 = 44,352
Dropout	Dropout	0	0
Output	Dense	2	288*2 + 2 = 578

### Convolutional neural network

A word or word embedding sequence is the CNN’s input. The input text is then transformed into a 2D matrix with a word embedding represented in each row [23]. The convolutional layer scans the input matrix using a collection of filters to extract pertinent n-grams (such as bi- or trigrams) of word embedding. The convolutional layer’s feature maps are subsequently subjected to an activation function, such as ReLU. The final classification decision is often made by feeding the output of the convolutional layer through one or more fully linked layers. To reduce a loss function, like cross-entropy, the network is trained using gradient descent and back propagation [24].

The mathematical representation for outcome of CNN for text is presented as


(14) \begin{equation*} y=f\left( Wx+b\right) \end{equation*}

Where y represents the network’s output, b symbolizes the bias term, f() exemplifies the activation function, W is the set of learnable filters, and x showcases the word embedding input matrix. Mathematically, the convolution operation itself is stated as follows:


(15) \begin{equation*} {y}_i=f\left(\sum \left(\mathrm{j}=1\ \mathrm{to}\ \mathrm{k}\right)\ {W}_j\ast{x}_{\left(i+j-1\right)}+b\right) \end{equation*}

Where x (i + j-1) denotes the input word embedding at position i + j-1, k represents the filter size, b represents the bias term, and y(i) exemplifies the output feature map at position i. W(j) is the weight at position j in the filter.

**Figure 3:**
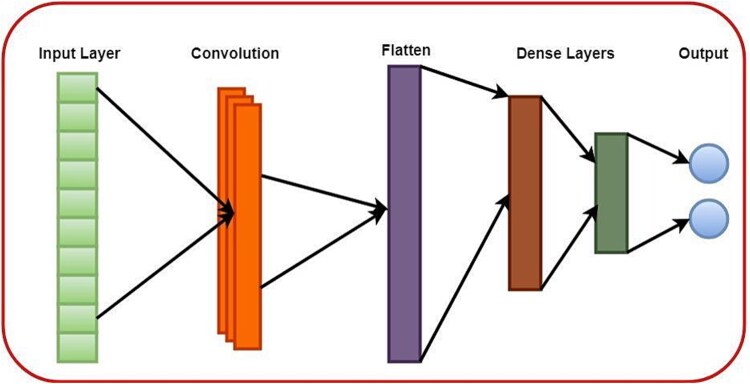
CNN graphical architecture.

Figure 3 resembles the CNN architecture that has been employed in this study for identification of BBB peptides.

Table 2 shows the parameters architecture for CNN architecture used in this study.

**Table 2:** CNN parametric architecture.

Layer	Type	No. of neurons	Total weights
Input	-	N (number of input features)	0
Conv 1D	Conv 1D	464	4640
MaxPooling1D layer	MaxPooling1D layer	464	0
Conv1D layer	Conv1D layer	464	1 938 128
MaxPooling1D layer	MaxPooling1D layer	464	0
Flatten	Flatten	14,848	0
Dense	Dense	96	1 425 504
Output	Dense	2	194

### Simple recurrent neural network

Recurrent neural networks (RNNs) address the limitation of FCNs in recognizing patterns at different sequence positions by sharing weights across time steps. This is achieved through a looping mechanism that allows the network to maintain a temporal state, effectively processing sequence vectors${x}_1,{x}_2\dots .{x}_n$. The RNN updates its hidden state ${a}_t$at each time step $t$ using the recurrence relation [25].

The recurrence relation is


(16) \begin{equation*} {a}_t={f}_a\left({\gamma}_t-1,{x}_t\right) \end{equation*}

Where $f$ is an activation function, $a$ is a set of shared parameters, and ${x}_t$ is the input at time step$t$. This structure enables RNNs to capture temporal dependencies and sequence patterns more effectively than FCNs.

Table 3 reflects the parametric architecture of Simple RNN for the BBB peptides identification.

**Table 3:** RNN parametric architecture.

Layer	Type	No. of neurons	Total weights
Input	-	N (153)	0
SimpleRNN	RNN	128	35,840
Dropout	Dropout	0	0
Dense	Dense	64	8256
Dropout	Dropout	0	0
Output	Dense	2	130

### Long short-term memory

The word embedding sequence serves as the LSTM’s input. At each time step t, the LSTM analyzes the input sequence and produces a hidden state vector, ${h}_t$. Based on the current input word embedding x_t and the prior hidden state vector${h}_{\left\{t-1\right\}}$, the hidden state vector ${h}_t$ is updated. The current hidden state vector ${h}_t$ is the output of the LSTM at each time step t. The last hidden state vector ${h}_T$ is often passed through one or more fully connected layers to create the LSTM’s final output [26]. To reduce a loss function, like cross-entropy, the network is trained using gradient descent and back propagation [23].

The output generated by an LSTM for a specific text input can be represented mathematically as follows:


(17) \begin{equation*} {h}_t=f\left(W{x}_t+U{h}_{\left\{t-1\right\}}\right)+b\Big) \end{equation*}

Where b is the bias term, $f\left(\right)$ is the activation function, such as the sigmoid or tanh function, W and U are the learnable weight matrices, and ${x}_t$ is the input word embedding at time step t. To reach the ultimate classification conclusion, additional layers, such as a fully linked layer, can be used to process the output of the LSTM at each time step.

**Figure 4:**
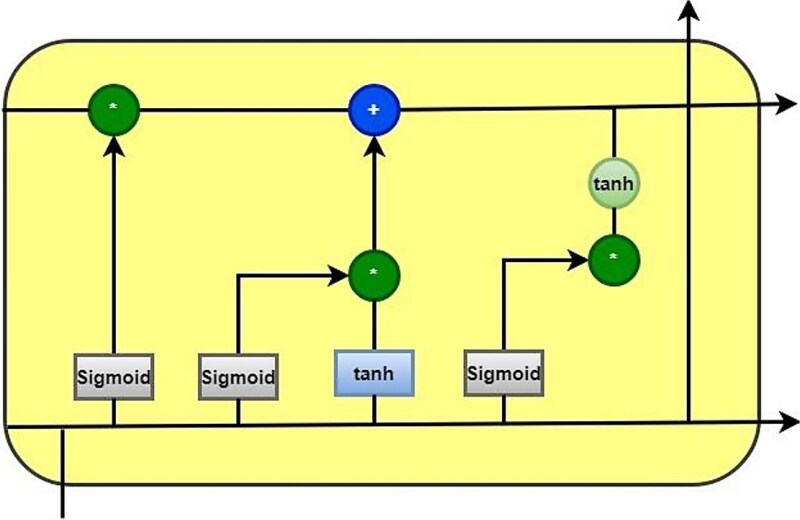
LSTM graphical architecture.

Figure 4 shows the employed LSTM architecture for prediction of BBB peptides.

Table 3 exemplifies the parametric values for LSTM architecture used in this study.

### Gated recurrent unit

The GRU receives a series of word embedding as input. At each time step t, the GRU analyses the input sequence and produces a hidden state vector${h}_t$. Based on the current input word embedding x_t and the prior hidden state vector${h}_{t-1}$, the hidden state vector ${h}_t$ is updated. The GRU features two gates that regulate how much of the prior hidden state should be retained and how much of the current input should be used. These gates are the update gate ${z}_t$ and the reset gate${r}_t$. The current hidden state vector ${h}_t$ is the output of the GRU at each time step t. The last hidden state vector ${h}_T$ is often passed through one or more fully linked layers to produce the GRU’s final output. To minimize a loss function, the network is trained via back propagation and gradient descent [18]. Table 5 shows the parametric architecture for GRU.

The mathematical expression for the output of a GRU given a specific text input is:


(18) \begin{equation*} {z}_t= sigmoid \ \Big({W}_z\ast{x}_t+{U}_z\ast{h}_{\left\{t-1\right\}}+{b}_z\Big) \end{equation*}

(19) \begin{equation*} {r}_t= sigmoid\ \Big({W}_r\ast{x}_t+{U}_r\ast{h}_{\left\{t-1\right\}}+{b}_r\Big) \end{equation*}

(20) \begin{equation*} h{\sim}_{\mathrm{t}}=\tanh \Big({W}_h\ast{x}_t+{r}_t\ast \Big({U}_h\ast{h}_{\left\{t-1\right\}}+{b}_h\Big)\Big) \end{equation*}

(21) \begin{equation*} {h}_t=\left(1-{z}_t\right)\ast{h}_{\left\{t-1\right\}}+{z}_t\ast h{\sim}_{\mathrm{t}} \end{equation*}

The learnable weight matrices are denoted by${W}_z$, ${U}_z$, ${W}_r$, ${W}_r$, ${W}_h$, and ${W}_h$; the bias terms are ${B}_z$, ${B}_r$, and ${B}_h$; the sigmoid function is Sigmoid; and the hyperbolic tangent function is Tanh. The input word embedding at time step t is denoted by${x}_t$. Each time step, the output of the GRU may be subjected to additional layers, such a fully connected layer, in order to determine the final classification decision.

**Figure 5:**
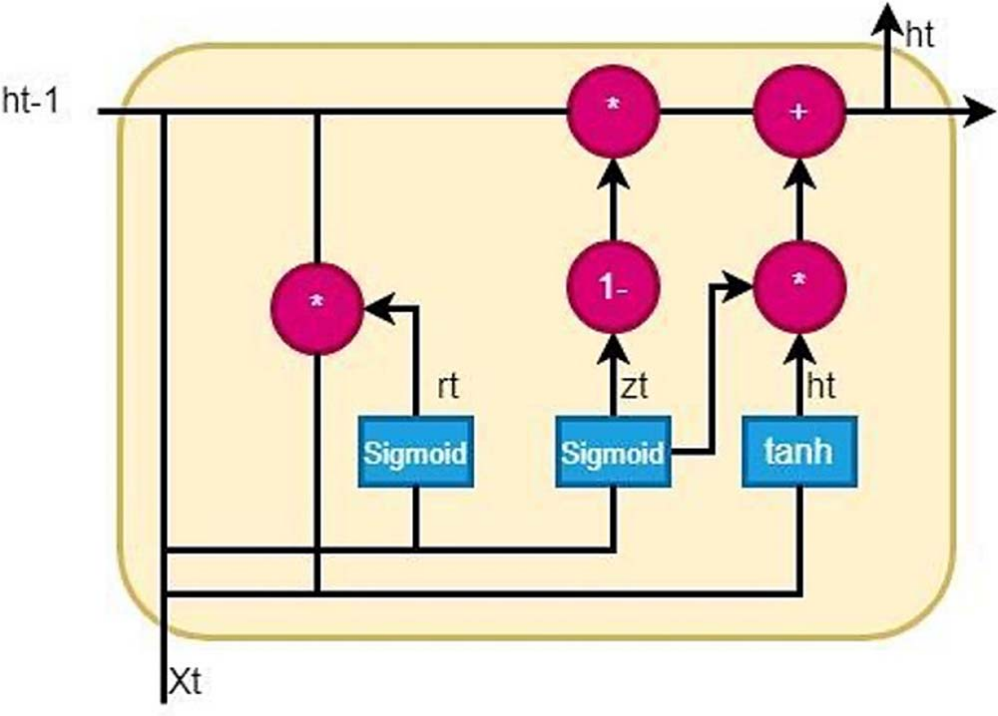
GRU graphical architecture.

Figure 5 demonstrates the GRU architecture used in this study for BBBPs identification.

Table 4 shows the parametric architecture for GRU architecture used in this study.

**Table 4:** LSTM parametric architecture.

Layer	Type	No. of neurons	Total weights
Input	-	N (153)	0
LSTM	LSTM	128	144 384
Dropout	Dropout	0	0
Dense	Dense	64	8256
Dropout	Dropout	0	0
Output	Dense	2	130

**Table 5:** GRU parametric architecture.

Layer	Type	No. of neurons	Total weights
Input	-	N (153)	0
GRU	GRU	256	315 648
Dropout	Dropout	0	0
Dense	Dense	128	32 896
Dropout	Dropout	0	0
Output	Dense	2	258

### Bidirectional long short-term memory

A BidirectionalLSTM (Bi-LSTM) is an extension of the standard LSTM that improves model performance on sequence classification problems. Unlike a traditional LSTM that processes input sequences in one direction (typically from past to future), a Bi-LSTM runs two LSTMs in parallel: one forward (from start to end) and one backward (from end to start). This bidirectional approach allows the model to capture information from both past and future contexts, leading to a richer understanding of the sequence data. The key difference from a regular LSTM is this dual processing, which enables Bi-LSTMs to achieve better performance on tasks where the context from both directions is important [27].

Table 6 exemplifies the Bi-LSTM parametric architecture for identification of BBB peptides.

**Table 6:** Bi-LSTM parametric architecture.

Layer	Type	No. of neurons	Total weights
Input	-	N (153)	0
Bidirectional	LSTM	128*2 = 256	295 936
Dropout	Dropout	0	0
Dense	Dense	64	16 448
Dropout	Dropout	0	0
Output	Dense	2	130

In the field of bioinformatics, supervised learning plays a key role in tasks like classifying genes and predicting protein structures. On the other hand, unsupervised learning helps researchers uncover hidden patterns in large sets of unlabeled genomic data. Transfer learning has also become invaluable, as it uses models trained on large biological datasets to tackle more specific tasks, especially when data is limited. Ensemble learning, which combines the strengths of multiple models, is particularly effective in areas like cancer classification, offering more reliable predictions. To enhance model performance, techniques like dropout (a form of regularization) and fine-tuning hyperparameters are commonly applied. For better interpretability, tools such as attention mechanisms and gradient-based visualizations allow researchers to understand how models make their decisions [28–31].

In this study, we focus on training deep neural networks, LSTM, Bi-LSTM, and CNN using supervised learning techniques. The training process is guided by gradient descent, ensuring the models learn effectively from the data.

### Pre-trained Evolutionary Scale Modeling 2.0

ESM represents a significant leap forward in protein sequence analysis by harnessing the capabilities of protein language models to derive both structural and functional insights from amino acid sequences. Traditionally, protein structure prediction has heavily relied on techniques like multiple sequence alignments (MSAs) or structural templates. However, ESM-2, the most advanced model of its kind, breaks away from these conventional methods. By training exclusively on the distributional patterns found within raw protein sequences, it achieves impressive accuracy in predicting protein structures, as well as classifying proteins based on their functional attributes. This novel approach highlights how understanding the inherent patterns in sequence data can unlock deeper insights into protein biology.

One of the standout features of ESM-2 is its scale and architecture. As the largest protein language model to date, it comprises an immense 15 billion parameters, making it an exceptionally powerful tool in bioinformatics research. ESM-2 utilizes transformer-based architectures, which are adept at capturing complex relationships between sequences. By training on extensive protein datasets, the model can recognize intricate sequence relationships, which in turn are indicative of three-dimensional structures. This allows it to go beyond surface-level analysis, enabling more sophisticated predictions and insights.

A key application of ESM-2 is in structure prediction, facilitated by a specialized framework known as ESMFold. Leveraging ESM-2’s deep sequence embeddings, ESMFold can perform end-to-end atomic-level structure predictions using just a single protein sequence as input [32]. This is a significant departure from other methods, such as AlphaFold, which often depend on MSAs for accurate structure prediction. By bypassing the need for homologous sequence alignments, ESMFold excels in scenarios where sequence homology is low, making it particularly effective for characterizing orphan proteins those for which no known relatives exist in current databases.

Beyond structure prediction, ESM-2’s capabilities extend to downstream tasks through fine-tuning. A scaled-down version of the model, comprising 35 million parameters, has been optimized for classification tasks. This involves predicting various attributes of proteins, such as their functional roles, cellular localization, and evolutionary families. Fine-tuning on specific tasks has demonstrated that scaling up the model leads to enhanced classification accuracy, underscoring that larger models can capture more nuanced and detailed biological information. As a result, this fine-tuned version is highly effective in annotating vast protein databases, particularly for sequences that are newly discovered and lack prior characterization.

Another critical advantage of ESM-2 is its ability to bridge the gap between sequence data and structural-functional insights. Unlike older models that rely on structural templates, ESM-2 leverages its understanding of sequence patterns to infer both structural and functional characteristics. This ability to predict a protein’s structural conformation directly from its sequence data without relying on MSAs or templates marks a paradigm shift in the field. For researchers working with uncharacterized proteins, ESM-2 opens new avenues for hypothesis generation and exploration, providing functional insights that were previously inaccessible.

By moving away from dependency on traditional alignment-based methods, ESM-2 sets a new standard for how protein data can be analyzed and understood. It holds promise not only for structural biology but also for broader applications such as drug discovery, functional annotation, and even the exploration of evolutionary relationships among proteins. This model exemplifies the growing role of AI in expanding our understanding of the protein universe, pushing the boundaries of what can be achieved with purely data-driven approaches [33].

The ESM-2 model was fine-tuned with a learning rate of `2e-5`, a batch size of 8, and 10 epochs. The number of labels is dynamically determined from the dataset. Regularization was applied with a weight decay of `0.01`. Evaluation and model saving occur after each epoch based on accuracy.

### Evaluation metrics

To assess the performance of the employed classifiers, a range of evaluation metrics are utilized, including accuracy score, specificity, sensitivity, and Matthews’s correlation coefficient. These metrics are used to evaluate the proposed model comprehensively. Out of all samples, the accuracy score indicates the total number of correct predictions from both classes [34, 35]. Specificity is a measure used in binary classification to evaluate the ability of a model to correctly identify true negatives from all negative samples. It quantifies the proportion of actual negatives that are correctly identified by the model as negatives [36]. The model’s sensitivity indicates how well it can find positive instances [37]. As MCC takes into account both classes, it is a trustworthy statistic even in the case of unbalanced data [38]. An astute MCC score will be generated by the model if it is able to identify both the positive and negative samples. The formulas for each discussed metric are provided.


(22) \begin{equation*} Accuracy=\frac{TP+ TN}{TP+ TN+ FP+ FN} \end{equation*}

(23) \begin{equation*} Specificity=\frac{TN}{TN+ FP} \end{equation*}

(24) \begin{equation*} Sensitivity=\frac{TP}{TP+ FN} \end{equation*}

(25) \begin{equation*} MCC=\frac{TN\times TP- FN\times FP}{\sqrt{\left( FP+ TP\right)\left( FN+ TP\right)\left( FP+ TN\right)\left( FN+ TN\right)}} \end{equation*}

A True Positive is a representation of the Peptides that the predictor correctly classified as being in the positive class. Peptides sequences that belong to the positive class but predicted as negative class by the predictor are referred to as false negatives. Conversely, False Positive denotes Negative samples that the predictor has tagged as Positive samples. A True Negative refers to samples that are correctly classified by the predictor as belongs to the negative class.

## Results

To assess predictor robustness, three types of stringent tests were used: the self -consistency test, the independent set test and five-fold CV.

### Self-consistency

The self-consistency test is a fundamental method used to assess the accuracy of a predictor. In this approach, the model was trained on the entire dataset without performing a train-test split to evaluate its performance. After training, all classifiers are tested to check if they successfully generated the model against the training dataset [39]. The self-consistency assessment demonstrates the model’s coherence with the dataset it was trained on, as it undergoes training and evaluation using the same dataset [40]. The results for self-consistency have been attached to the supplementary material.

### Independent testing

Independent testing is another way for evaluating a predictor’s performance with unknown data. For this examination, the data is typically separated into two parts. The first section, which accounts for 77% of the total dataset, is used for training. This portion provides the model with input–output pairs to help it learn accurately. The remaining 23% is used to evaluate the predictor’s accuracy. During this testing step, the predictor is just shown input features; the class name is not disclosed. Based on data that was unavailable during the training phase, the predictor produces predictions [41].

Table 7 shows that the Fine-tuned ESM 2.0 demonstrates exceptional performance, with accuracy score of 0.843. The obtained outcome from independent set test shows that the model functions effectively on data that was not seen during the training.

**Table 7:** Results from the independent set.

Classifier	Accuracy	Sensitivity	Specificity	MCC
FCN	0.786	0.752	0.821	0.574
CNN	0.806	0.772	0.842	0.615
LSTM	0.756	0.728	0.789	0.513
GRU	0.75	0.727	0.779	0.502
RNN	0.776	0.772	0.779	0.55
Bi-LSTM	0.745	0.702	0.79	0.493
Fine-tuned ESM 2.0	0.843	0.824	0.861	0.686

**Figure 6:**
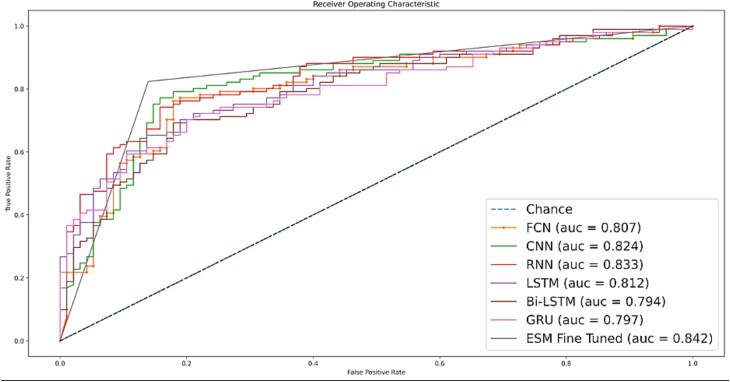
ROC curve for independent set test.

Figure 6 visualizes the ROC for independent-set testing, the fine-tuned ESM 2.0 model outperforms other predictors.

### Five-fold cross-validation

CV is a rigorous test that is utilized on every sample [42]. The process involves dividing the data into five equal folds. In each iteration, four of these folds are used for training, while one-fold is set aside for testing. This process is repeated until every fold has been used to generate predictions on every sample. The accuracy of each fold is calculated individually, and at the end, the average accuracy is computed. This method ensures that each sample of data is independently examined and trained, providing a thorough evaluation of the model’s performance. Table 3 displays the outcomes for every classifier.

Among the utilized classifiers, the LSTM outperformed the others in five-fold CV test. It shows excellent accuracy and MCC score of 0.756 and 0.511, correspondingly. Table 8 shows how the results of the five-fold CV provide useful information into the predictor’s effectiveness.

**Table 8:** Results using five-fold CV.

Classifier	Accuracy	Sensitivity	Specificity	MCC
FCN	0.758	0.722	0.79	0.514
CNN	0.747	0.725	0.771	0.5
RNN	0.749	0.723	0.772	0.5
LSTM	0.756	0.725	0.785	0.511
Bi-LSTM	0.753	0.72	0.783	0.504
GRU	0.746	0.69	0.8	0.493

**Figure 7:**
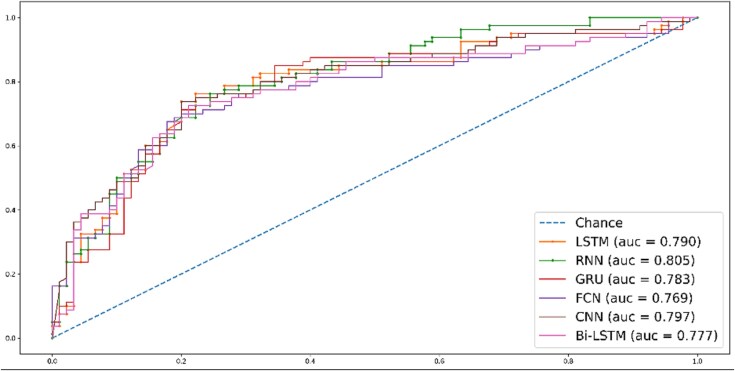
ROC graph using five-fold CV*.*

Figure 7 graphically represents the findings from the five-fold CV. The figure showcases that, RNN has outperformed other classifiers by attaining the AUC score of 0.805.

**Figure 8:**
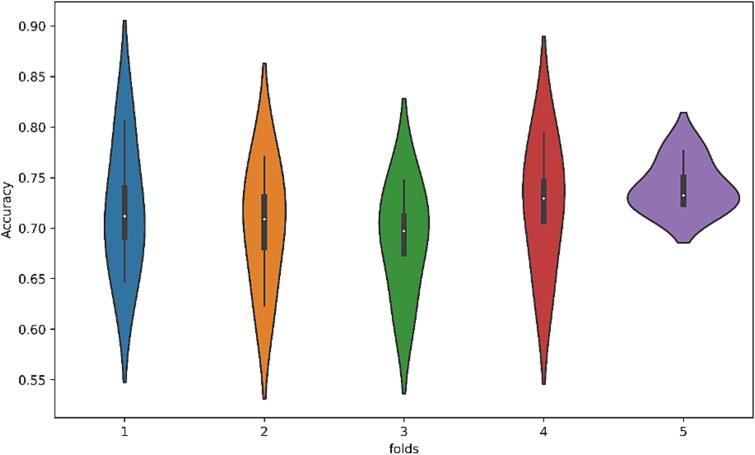
Violin plot using five-fold.

To graphically illustrate the distributions of multiple groups, the violin plot syndicates the qualities of boxplots and kernel density charts. Figure 8 demonstrates a violin chart illustrating the accuracy of five-fold CV findings.

Table 9 showcases comparative analysis for BBB peptides identification. The proposed approach BBB-PEP-ESM surpasses previous state-of-the-art benchmark studies. The suggested study’s independent set testing yielded these results.

**Table 9:** Comparison with benchmark studies.

Study	Accuracy	Sensitivity	Specificity	MCC
BBPpred [7]	66.67	65.66	67.68	33.34
B3Pred [8]	67.68	64.65	70.71	35.42
BBBPredict [9]	77.27	77.78	767.7	54.55
BBB-PEP-prediction [10]	82.4	83.1	91.1	66.3
BBB-PEP-ESM	84.3	86.1	82.4	68.6

Table 10 provides the list of BBBP’s identification using proposed method. It shows the peptide sequences, trivial names, and their identification status.

**Table 10:** List of peptides identification.

Sr	Peptide	Name	Status	Probability	Source
1	BBBP	JMV 2012	Identified	0.91	[12]
2	BBBP	TAPS	Identified	0.93	[12]
3	BBBP	PhrCACET1	Identified	0.91	[12]
4	BBBP	D3D3	Identified	0.92	[12]
5	BBBP	MVIIA-a	Identified	0.9	[11]
6	BBBP	Exendin-1	Identified	0.78	[11]
7	BBBP	Neuropeptide Y	Identified	0.86	[11]

## Discussion

An in-silico technique has been used in this study for BBB peptides prediction. Initially, human-engineered features were used for peptides, with positional and composition variation features used to convert raw sequences into vector representation. The resultant vector was high-dimensional; thus, dimensionality was reduced by utilization of Raw, Hahn, and central-based statistical moments. This study gathered more information regarding the characteristics of peptides than earlier studies. State-of-the-art Deep learning architectures such as FCN, CNN, LSTM, and GRU were used to detect the BBB peptides. Keras Tuner was used to discover the ideal parameters for accuracy enhancement in FCN and CNN. The employed classifiers effectively discriminated between the positive and negative class by obtaining the high coefficients shown by feature space for BBB peptides prediction. To evaluate the predictor’s performance, three testing methods were employed: self-consistency, independent set testing, and five-fold CV. Additionally, a pre-trained protein language model, ESM 2.0, was fine-tuned using a benchmark dataset to achieve better results than existing benchmark studies. The ESM 2.0 and CNN classifier consistently outperformed existing approaches in independent set test by obtaining accuracy score of 0.842 and 0.806 respectively. ESM 2.0, as a pre-trained model, has outperformed previous studies. Its capacity to handle varied Amino Acid patterns and record context-specific representations makes it resilient. The excellent results of ESM 2.0 originate from its deep architecture, large training data, and integration of cutting-edge transformer-based approaches, confirming its effectiveness in obtaining top-tier performance in performed studies. CNN attained ACC, SPE, SEN and MCC scores of 0.806, 0.842, 0.772, and 0.615 respectively in independent set test. In the independent set our previous study [9], showcases the highest accuracy score of 0.824. The fine-tuned model ESM 2.0 acquired an improved Accuracy score of 0.843. In addition, the table 10 highlights the sample BBBP’s identified using BBB-PEP-ESM. When compared to other feature computation methodologies, the feature vector utilized in this study are vigorous and rigorous in capturing the qualities of sequences. Overall, the results, notably in independent set test, indicate that the predictor has a high level of generalization potential.

### Boundary visualization

In the following section, we will guide you through the process of visualizing decision boundaries. Decision boundary is a line that divides sequences from individual class. Boundary visualization from individual deep classifier and raw data sequence visualization are two of the visualization approaches utilized in this study.

**Figure 9:**
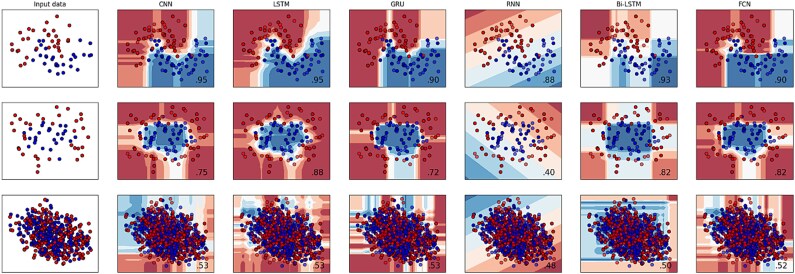
Boundary visualization for each classifier.

Figure 9 depicts the border visualization for each deep classifier, demonstrating the categorization of samples from both classes by establishing separate discrimination boundaries. Individual classifier created its own class discriminating space for both classes after passing through heterogeneous classifiers.

**Figure 10:**
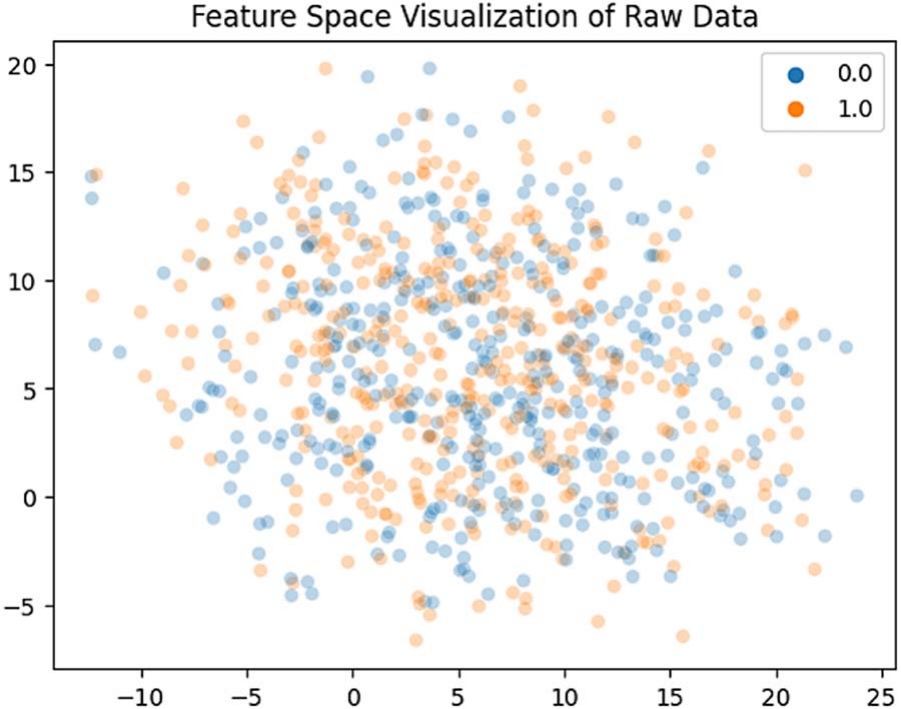
Feature space visualization.

The raw feature space representation, displayed in Fig. 10, illustrates the separateness of the raw data. The feature space presentation produces noteworthy results, demonstrating that the calculated features on BBB peptides have a substantial amount of discriminatory information. The presented study’s classifiers effectively distinguish between two unique clusters in the data, each of which reflects a different class.

## Conclusion

This research was carried out for computational identification of BBB peptides. The use of BBBPs can allow drugs to be transported into the brain, which creates new opportunities for the progress of therapies focused at treating CNS problems. This significance motivates the computational identification of these peptides. In this study, various feature computing techniques were used to extract hidden information inside sequences. For peptide sequences, feature vectors such as AAPIV, RAPPIV, PRIM, RPRIM, and FV have been computed. These feature computation techniques have provided important insights into protein characteristics. State-of-the-art Deep learning architectures such as FCN, CNN, RNN, LSTM, Bi-LSTM and GRU were employed to detect the BBB peptides. Keras Tuner was used to determine the best parameters to enhance accuracy. The utilized classifiers commendably discriminated between the two classes, for BBB peptides identification. To evaluate the performance, testing such as self-consistency, independent set testing, and five-fold CV have been employed. In addition, a pre-trained protein language model ESM 2.0 has been fine-tuned on benchmark dataset. In an independent set test, a fine-tuned ESM 2.0 has surpassed other classifiers with accuracy score of 0.843. ESM 2.0, as a pre-trained model, has proven its usefulness by outperforming the existing studies. Its ability to handle diverse Amino Acid patterns and capture context-dependent representations makes it robust. ESM 2.0’s impressive results stem from its deep architecture, extensive training data, and the integration of state-of-the-art transformer-based methodologies, demonstrating its effectiveness in achieving top-tier performance in conducted study. In the independent set test, ESM 2.0 achieved accuracy, sensitivity, specificity and MCC scores of 0.843, 0.861, 0.824, and 0.686, respectively. This work investigates the identification of BBB peptides, and in the future, the BBB-PEP-Prediction can be employed for BBB peptide identification. It is worth mentioning, however, that this study has a disadvantage in that we have used the previous research dataset for experiment purposes. In the future work, we will increase the dataset to discover more peptides. Our research has made significant strides in accurately identifying BBB peptides. The model we developed, BBB-PEP-Prediction, outperforms previous approaches in terms of accuracy. However, one limitation is that we relied on existing datasets from earlier studies. This can limit the diversity of the data we used and might introduce biases, which could affect how well our model generalizes to new, unseen data. To address this, we plan to expand our dataset in the future by including a wider variety of peptide sequences. This will help improve the model’s reliability and make it more effective for real-world applications.

**Key Points**

- BBBPs enhance drug delivery to the brain, overcoming the blood–brain barrier, which obstructs nearly 98% of small molecule drugs and almost all large molecule drugs.
- The study uses relative positions, reverse positions, and statistical moment-based features on benchmark dataset to improve BBB peptide identification.
- Six deep learning classifiers (FCN, CNN, RNN, LSTM, Bi-LSTM, and GRU) were utilized for effective BBB peptide classification.
- The Deep models have evaluated on self-consistency, independent set testing, and five-fold CV.
- The pre-trained protein language model ESM 2.0 was fine-tuned on a benchmark dataset and excelled in, independent set testing by surpassing existing benchmark studies in ACC, SPE, SEN, and MCC.

## Supplementary Material

Supplementary_Material_bbaf066

## Data Availability

The utilized benchmark dataset and code in this study is provided is uploaded on GitHub https://github.com/Ansar390/BBB_DEEP_ESM2.0 accessed on 2-2-2025.
