# Supplementary material for: ESM-BBB-Pred: a fine-tuned ESM 2.0 and deep neural networks for the identification of blood–brain barrier peptides
Source: Brief Bioinform. 2025 Feb 23;26(1):bbaf066. doi: 10.1093/bib/bbaf066 (PMC12079436; doi:10.1093/bib/bbaf066)
Supplement: Supplementary_Material_bbaf066 [file supplementary_material_bbaf066.docx]

The supplementary material is attached to the GitHub link attached below.

<https://github.com/Ansar390/BBB_DEEP_ESM2.0>
